# Comprehensive evaluation of functional vision, quality of life, and cognitive ability in pediatric uveitis

**DOI:** 10.1186/s12886-023-03117-7

**Published:** 2023-09-18

**Authors:** Wenjuan Wan, Zhijun Chen, Yan Xun, Kun Yi, Ying Zhu, Yanlin Pu, Guannan Su, Chunjiang Zhou, Yao Wang, Peizeng Yang

**Affiliations:** 1 The First Affiliated Hospital of Chongqing Medical University, Chongqing Key Lab of Ophthalmology, Chongqing Branch of National Clinical Research Center for Ocular Diseases, Chongqing Eye Institute, Chongqing, P. R. China; 2 The First Affiliated Hospital of Chongqing Medical University, Youyi Road 1, 400016 Chongqing, P.R. China

**Keywords:** Pediatric uveitis, Visual function, Quality of life, Cognition, Anxiety, Depression.

## Abstract

**Background:**

Pediatric uveitis may cause severe impairment of vision in children and affect their quality of life as well as cognitive ability. This study aims to evaluate the functional vision, visual-related and health-related quality of life, and cognitive ability in pediatric uveitis.

**Methods:**

Children with uveitis aged 5–16 years old completed six validated instruments to assess functional visual ability with Cardiff Visual Ability Questionnaire for Children (CVAQC), vision-related quality of life with Impact of Vision Impairment for Children (IVI-C), health-related quality of life with Pediatric Quality of Life Inventory (PedsQL), cognitive ability with Chinese Wechsler Intelligence Scale for Children (C-WISC), and depression and anxiety evaluation with Hospital Anxiety and Depression Scale (HAD).

**Results:**

The CVAQC, IVI-C, and PedsQL scores of pediatric uveitis were significantly lower than that of normal levels. Full-scale intelligence quotient (IQ) and performance IQ were significantly lower in pediatric uveitis patients with impaired vision in their best eye (visual acuity < 0.3) compared to those with a vision equal to or better than 0.3. Verbal IQ was significantly lower in male pediatric uveitis patients with impaired vision compared to those with a vision equal to or better than 0.3. Additionally, parents of pediatric uveitis patients with impaired vision generally had lower educational levels than parents of those with a vision equal to or better than 0.3.

**Conclusions:**

Impaired vision caused by pediatric uveitis has a significant impact on children’s functional visual ability and quality of life. The development of cognitive function in pediatric uveitis is also significantly hindered.

## Introduction

Uveitis is an ocular inflammatory disease in the middle layer of the eye. It can be caused by infection (infectious uveitis) or immune response (noninfectious uveitis). Noninfectious uveitis mostly arises from multifactorial etiologies such as genetic factors, environmental stimulation, and abnormal immune cell activation [1, 2]. Pediatric uveitis accounts for approximately 5–10% of all patients with uveitis, and most pediatric uveitis cases (approximately 90%) are noninfectious [3]. It is usually asymptomatic, chronic, persistent and recurrent [1]. In addition, the clinical presentations of pediatric uveitis such as eye redness, blurry vision and pain vary greatly in different patients. The diagnosis of pediatric uveitis may be delayed due to the absence of obvious symptoms and difficulties in performing comprehensive eye examinations. With the progression of the disease, it is often associated with many ocular complications including cataract, band keratopathy, synechiae formation, macular edema and even hypotony [4]. Severe ocular complications can lead to irreversible morphological damage and visual disability. There are many challenges in the management of pediatric uveitis, including delayed diagnosis, risk of amblyopia, and limited treatment options [5]. Currently, the treatment of pediatric uveitis is to achieve remission of intraocular inflammation and reduce severe ocular complications and lifelong burden of vision loss on the family [2, 6].

Good vision is critical for children’s daily activities such as reading, writing, and social activities. Poor visual outcomes can produce a negative effect on quality of life (QoL) in children with uveitis, and severely impair children’s social activities, cognitive ability, and psychological and physical well being [7]. The vision-related QoL (VR-QoL) in children with uveitis may be further aggravated due to persistent and chronic inflammation, corticosteroid administration, and delayed use of agents for modifying systemic diseases [6]. Some studies have investigated the QoL in children with uveitis and found that measurement of VR-QoL is important for evaluating the vision related life quality of pediatric uveitis [8–10]. It has been reported that uveitis in adults with impaired vision has worse QoL and high rates of anxiety and depression [11, 12]. However, few studies have addressed these issues in the quality of life, cognitive function as well as emotional statements in pediatric uveitis. A comprehensive approach that includes multiple aspects of disability may improve the assessment of outcomes in children with uveitis.

Here we performed a study to evaluate functional visual ability, vision-related quality of life (VR-QoL), health-related quality of life (HR-QoL), cognitive ability, as well as depression and anxiety evaluation in pediatric uveitis. The results showed that impaired vision caused by pediatric uveitis has a significant impact on their behavior, cognitive and emotional development.

## Methods

### Patients

This prospective study was performed in accordance with the tenets of the Declaration of Helsinki, and was approved by the Ethics Committee of the First Affiliated Hospital of Chongqing Medical University (Approval number: 2019 − 210). Patients with pediatric uveitis who visited the uveitis center in the First Affiliated Hospital of Chongqing Medical University between June 22, 2021 and September 27, 2021 were recruited. Inclusion criteria included: (1) a diagnosis of pediatric noninfectious uveitis; (2) age between 5 and 16 years at diagnosis. Exclusion criteria included: (1) significant co-morbidity unrelated to uveitis (i.e. sickle cell anemia) affecting QOL and function; (2) major developmental disorders (i.e. cerebral palsy, mental retardation); (3) major emotional disorders unrelated to uveitis (i.e. obsession, phobias); (4) other ocular disorders (i.e. pathological myopia) unrelated to uveitis; (5) patients who were unable to communicate in Chinese or underwent surgical intervention within 1 month before or after completing questionnaires were excluded from the study. The medical records of all children were screened to identify those who met inclusion criteria. Age-appropriate information materials and questions were provided.

All patients’ clinical data such as age, gender, racial or ethnic background, ocular and systemic manifestations, the age at uveitis onset, disease course, ocular complications secondary to uveitis, and previous and current medical treatments (including surgical interventions) were reviewed and recorded. The systemic medications (such as glucocorticoids, immunosuppressants, etc.) used currently or previously, and topical medications were also reviewed. Patients with pediatric uveitis and their parents had given written consent before their inclusion in the study. The parental education backgrounds of patients were recorded.

### Ophthalmological examination

A complete ophthalmic evaluation including best corrected visual acuity (BCVA), refraction, intraocular pressure (IOP), slit-lamp inspection, ophthalmoscopy of the fundus was performed in all the patients included in this study. Fundus fluorescein angiography (FFA), ocular B-ultrasound, and optic coherence tomography (OCT) were performed in 104, 83 and 113 cases respectively. The BCVA was assessed and the better one was recorded with a logarithm of the minimum angle of resolution score using the standard logarithmic visual acuity chart. Moderate and severe visual impairment was defined as BCVA < 0.3 following the International Council of Ophthalmology (ICO) 2002 definitions [13].

### Questionnaire on functional vision

The Cardiff Visual Ability Questionnaire for Children (CVAQC) was used to evaluate functional vision in children. CVAQC is a self-report tool including 25 questions, which cover the areas of education, near/distance vision, social interaction, entertainment, and sports. Each question was answered based on a 4-point scale (0–3 points), resulting in total scores ranging from 0 (normal visual ability) to 75 (severe visual impairment) [14, 15].

### Questionnaire on VR-QoL

The Impact of Vision Impairment for Children (IVI-C) tool was used to evaluate VR-QoL in children aged 8 to 16 years [16]. The IVI-C included 24 questions, covering areas of school, mobility, social interaction, and emotion. For each question, 5 options (scored 0–4) and an additional option of “no, for other reasons” (no value) were available for selection. The total scores ranged from 0 (lowest VR-QoL) to 96 (normal VR QoL).

### Questionnaire on HR-QoL

Age-specific versions of the PedsQL Inventory (www.pedsql.org) were used to evaluate HR-QoL in children of 5 years and older, based on physical and emotional state as well as social and school life [17, 18]. The condition of the children was also reported by their parents through questionnaires (“parental report”), which include 21–23 questions for children aged 5–7, 8–12, and 13–16 years. The self-administered the questionnaire (PedsQL administration guidelines) was given to all the children tested with answers on 0–4 Likert scale. The PedsQL scores were calculated according to the scoring instructions. When blank items occurred, the denominator was adjusted by using the number of completed items instead of total items. Questionnaires were removed from the analysis when ≥ 50% of the items were left blank. Scores ranged from 0 (lowest HR-QoL) to 100 (normal HR-QoL).

### Evaluation on cognitive ability

Chinese Wechsler Intelligence Scale for Children (C-WSIC), which is designed to measure intelligence scores [19], was used to determine whether the cognitive abilities were affected in children with uveitis. C-WSIC contained ten core subtests and five additional subtests, which were summed into a four-index score containing Verbal Comprehension Index (VCI) and Perceptual Reasoning Index (PRI). The VCI included the Vocabulary, Similarities, and Comprehension subtests; the PRI included the Block Design, Picture Concepts, and Matrix Reasoning subtests. All four of the index scores were included in the calculation of full-scale intelligence quotient (IQ) (ranging from 40 points to 160 points).

Before initiation of the study, the testers were trained by professional institutions, and the consistency of their tests and scores was confirmed. All the tests were performed by professionally trained research members (Y. Z, Z. J. C). The test and scoring were carried out in strict accordance with the requirements of the test manual.

### Evaluation on anxiety and depression

The Hospital Anxiety And Depression Scale (HADS) was used to evaluate the severity of anxiety and depression, which included 7 anxiety items and 7 depression items [20]. Each item was scored by a 4-point (0–3) scale, resulting in a total score ranging from 0 to 21 for anxiety or depression. The state of anxiety or depression was defined for either subscale as follows: normal, 0 to 7; suggestive of the presence of mood disorder, 8 to 10; and probable presence (‘caseness’) of the mood disorder, 11 or higher. For patients with illiteracy or poor vision, the HADS and possible responses were read to the patients.

### Data analysis

Data analysis was performed using SPSS version 25.0 (SPSS Inc., Chicago, IL, USA). Descriptive statistics was applied throughout the study. The correlation analysis was performed using Spearman test. The rank-sum test or independent t test was used to compare variables. P values < 0.05 were considered statistically significant.

## Results

### General information

**Fig. 1:**
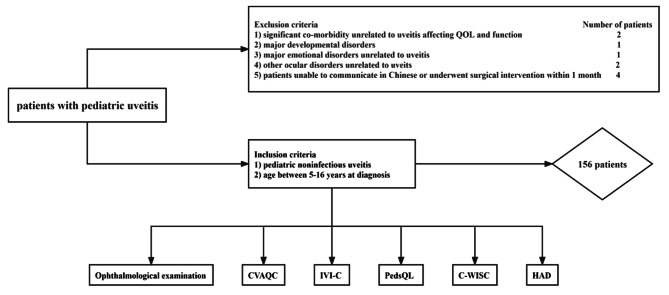
A flow chart of research inclusion and exclusion processes. CVAQC: Cardiff Visual Ability Questionnaire for Children, IVI-C: vision-related quality of life with Impact of Vision Impairment for Children, PedsQL: health-related quality of life with Pediatric Quality of Life Inventory, C-WISC: cognitive ability with Chinese Wechsler Intelligence Scale for Children, HAD: depression and anxiety evaluation with Hospital Anxiety and Depression Scale

A total of 156 patients with pediatric uveitis were enrolled according to the inclusion and exclusion criteria. The flow chart of this study is shown in Fig. 1. The demographic and clinical characteristics of 156 patients as stated above are listed in Table 1. Seventy-seven (49.4%) patients were female and seventy-nine (50.6%) were male. The mean age of all patients was 9.52 ± 3.30 years, and the mean age at diagnosis was 7.35 ± 3.12 years. The median BCVA at study participation was 0.8 (IQR, 0.3-1.0). The median daily dose of systemic glucocorticoids and cyclosporine were 0.392 mg/kg per day (IQR, 0.283–0.567), 3.329 mg/kg per day (IQR, 2.833–3.873). Ocular complications occurred in 156 patients, including cataract (61, 39.10%), glaucoma (20, 12.82%), band keratopathy (58, 37.18%), hypopyon (1, 0.64%), synechia (88, 56.41%), macular edema identified clinically by FFA or OCT (37, 23.72%), papilledema (54, 34.62%), and vitreous opacities (30, 19.23%). Patients were also associated with other systemic signs, including ulcer of mucous membrane and genitalia (7, 4.49%), joint pain (15, 9.62%), erythema of skin (2, 1.28%), leucoderma (2, 1.28%), white hair (2, 1.28%) and tinnitus (2, 1.28%).

**Table 1:** Demographic and clinical characteristics, associated conditions in study participants

	Age 5–7 years	Age 8–12 years	Age 13–16 years	All age groups
	n = 49	n = 73	n = 34	n = 156
**Age at study participation**				
Mean (SD)	6.72 (0.87)	10.52 (1.58)	14.27 (0.98)	9.52 (3.30)
**Age at diagnosis**				
Mean (SD)	5.82 (1.13)	8.33 (2.49)	9.54 (4.14)	7.35 (3.12)
**BCVA (LogMAR) at participation**				
Median (IQR)	0.75 (0.375-1.0)	0.8 (0.4-1.0)	0.55 (0.1-1.0)	0.8 (0.3-1.0)
**Number of eye surgery**				
Median (IQR)	0 (0-1.25)	0 (0–2.0)	0 (0-0.25)	0 (0–1.0)
**General medication**				
Current daily average dose of systemic glucocorticoids (mg/kg per day)				
Median (IQR)	0.354 (0.236–0.472)	0.386 (0.297–0.593)	0.412 (0.294–0.490)	0.392 (0.283–0.567)
Current daily average dose of cyclosporine (mg/kg per day)				
Median (IQR)	2.948 (2.358–3.538)	3.264 (2.967–4.006)	3.529 (2.941–3.922)	3.329 (2.833–3.873)
**Ethnic minority**			n	%
Tujia			6	3.85
Miao			3	1.92
Other minorities			17	10.90
Unknown			2	1.28
**Uveitis subtype**			n	%
Behcet’s disease			2	1.28
Vogt-Koyanagi Harada’s disease			3	1.92
Juvenile idiopathic arthritis			4	2.56
Blau Syndrome			4	2.56
Idiopathic pediatric uveitis			143	91.67
**Ocular complications**			n	%
Complicated cataract			61	39.10
Secondary glaucoma			20	12.82
Band keratopathy			58	37.18
Hypopyon			1	0.64
Synechia			88	56.41
Macular edema			37	23.72
Papilledema			54	34.62
Vitreous opacities			30	19.23
**Associations**				
Ulcer of mucous membrane and genitalia			7	4.49
Joint pain			15	9.62
Erythema of skin			2	1.28
Leucoderma			2	1.28
White hair			2	1.28
Tinnitus			2	1.28
Other exceptions			14	8.97

### Evaluation of functional visual acuity

All the children included in this study completed the CVAQC. The median of the CVAQC scores was 18.00 (IQR, 6.00–28.00), suggesting a moderate impairment of FVA in these patients [14, 15] (Table 2). In addition, Spearman rank correlation test showed no significant association of the CVAQC scores with age or the parameters investigated including BCVA, undergoing of surgery, medication used, ocular complications, or systemic signs (Table 3).

**Table 2:** Scores for functional visual ability (FVA), vision-related quality of life (VR-QoL) and health-related quality of life ( HR-QoL) reported by children according to age and parents

			PedsQL	
	CVAQC	IVI-C	Self-report	Parental report
	Median (IQR)			
	n = 156	n = 107	n = 156	n = 156
All age groups	18.00 (6.00–28.00)	80.00 (69.50–86.50)	76.00 (65.00–86.00)	73.00 (62.00–87.00)
	n = 49		n = 49	n = 49
Age 5–7 years	17.50 (4.75–25.25)	/	81.00 (57.00–90.00)	76.50 (68.75-90.00)
	n = 73	n = 73	n = 73	n = 73
Age 8–12 years	18.00 (8.00–28.00)	80.00 (66.00–87.00)	77.00 (65.00–84.00)	73.00 (59.00–85.00)
	n = 34	n = 34	n = 34	n = 34
Age 13–16 years	21.50 (3.00-33.50)	80.50 (72.25–84.75)	70.50 (61.25–85.25)	64.50 (54.75–87.25)

Possible CVAQC scores (FVA) range from 0.0 (higher FVA) to 75.0 (lower FVA). IVI-C scores range from 0 to 96 (severe reduction to normal VRQoL). PedsQL scores range from 0 to 100 (severe reduction to normal HR-QoL). Children reported markedly reduced FVA and VR-QoL. All HR-QoL scores were signifificantly reduced as reported by both children and parents

**Table 3:** Correlations between the scores of CVAQC, IVI-C, PedsQL, C-WISC, HAD, CES-DC and clinical characteristics

		CVAQC	IVI-C	PedsQL self-report	PedsQL parental report	C-WISC VIQ	C-WISC PIQ	C-WISC FIQ	HAD for anxiety	HAD for depression	CES-DC
BCVA	SRRC	0.205	-0.261	0.141	0.153	-0.042	-0.120	-0.097	0.065	0.082	-0.178
	P value	0.133	0.118	0.306	0.266	0.771	0.402	0.499	0.663	0.582	0.231
Number of undergoing surgery	SRRC	0.058	0.223	-0.041	-0.028	0.001	0.193	0.085	0.005	0.173	0.099
	P value	0.677	0.184	0.768	0.837	0.993	0.175	0.554	0.972	0.245	0.510
Age at study participation	SRRC	0.122	-0.027	0.075	0.285	0.099	-0.333	-0.105	0.000	-0.054	0.103
	P value	0.376	0.876	0.587	§ 0.035	0.491	§ 0.017	0.463	1.000	0.719	0.493
Age at diagnosis	SRRC	0.170	-0.176	0.042	0.255	0.230	-0.284	-0.021	-0.30	-0.70	0.095
	P value	0.213	0.296	0.759	0.061	0.104	§ 0.044	0.885	0.843	0.640	0.525
**General medication**											
Current daily dose of systemic glucocorticoids (mg)	SRRC	-0.153	-0.244	0.037	-0.001	0.161	0.203	0.149	-0.038	-0.012	0.080
	P value	0.263	0.146	0.788	0.996	0.259	0.153	0.298	0.802	0.937	0.595
Current daily dose of cyclosporine (mg)	SRRC	-0.138	0.205	-0.132	-0.119	0.222	-0.173	0.042	0.088	0.166	0.309
	P value	0.314	0.223	0.337	0.387	0.117	0.225	0.767	0.558	0.264	§ 0.034
**Ocular complications**											
Complicated cataract	SRRC	-0.041	0.166	0.092	0122	-0.177	0.139	-0.045	-0.008	0.097	0.137
	P value	0.769	0.326	0.505	0.377	0.215	0.329	0.751	0.956	0.516	0.357
Secondary glaucoma	SRRC	0.092	0031	-0.098	0.103	0.175	0187	0.208	-0.023	0.004	0.069
	P value	0.503	0.857	0.476	0.456	0.220	0.190	0.143	0.877	0.979	0.644
Band keratopathy	SRRC	-0.201	-0.068	-0.172	0.121	-0.015	-0.042	-0.031	-0.075	0.218	0.110
	P value	0.142	0.687	0.210	0.378	0.917	0.771	0.827	0.618	0.141	0.462
Synechia	SRRC	-0.073	0.181	0.081	0.098	0.029	0.105	0.062	-0.054	0.056	0.278
	P value	0.598	0.285	0.556	0.478	0.840	0.464	0.665	0.716	0.708	0.058
Macular edema	SRRC	0.129	0.111	0.115	0.180	-0.083	-0.185	-0.145	0.097	0.019	-0.015
	P value	0.349	0.514	0.405	0.188	0.564	0.193	0.309	0.517	0.897	0.918
Papilledema	SRRC	0.029	0.019	0.056	0.125	0.122	-0.264	-0.087	-0.173	-0.196	-0.149
	P value	0.832	0.911	0.684	0.362	0.395	0.061	0.542	0.244	0.187	0.318
Vitreous opacities	SRRC	-0.088	-0.056	0.126	0.033	-0.014	0.065	-0.071	-0.120	0.036	0.080
	P value	0.521	0.743	0.358	0.812	0.921	0.649	0.621	0.422	0.812	0.591
**Systemic complications**	SRRC	-0.122	0.113	-0.355	-0.031	-0.177	-0.046	-0.147	-0.067	-0.202	-0.038
	P value	0.375	0.504	§ 0.008	0.822	0.215	0.751	0.304	0.655	0.173	0.802

Spearman rank correlation test, *P* < 0.05;

CVAQC = Cardiff Visual Ability Questionnaire for Children, IVI-C = Impact of Vision Impairment for Children; PedsQL = ediatric Quality of Life Inventory; C-WISC = Chinese Wechsler Intelligence Scale for Children; HAD = Hospital Anxiety and Depression Scale; CES-DC = Center for Epidemiological Studies of Depression scale for Children;

§Statistically significant difference

### Evaluation of VR-QoL

Patients with pediatric uveitis aged 8–16 years enrolled completed the IVI-C tool. The median of the IVI-C score was 80.00 (IQR, 69.50–86.50), suggesting that the patients had markedly reduced VR-QoL [16, 21] (Table 2). Spearman rank correlation test showed no significant association of the CVAQC scores with any clinical parameters (Table 3).

### Evaluation of HR-QoL

PedsQL self-report questionnaires were completed by 156 patients. The median PedsQL self-report score was 76.00 (IQR, 65.00–86.00), which was lower than that of normal level [22, 23] (Table 2). Spearman rank correlation test showed that the PedsQL self-report scores were significantly associated with systemic signs (Spearman’s r correlation coefficient, r=-0.355; p = 0.008), but not associated with any other clinical parameters (Table 3).

PedsQL parental questionnaires were completed by 156 patient’s parents. The median PedsQL parental report score about the children was 73.00 (IQR, 62.00–87.00), significantly lowering than normal level (Table 2). Spearman rank correlation test showed that the parental report scores were significantly associated with the age at study participation (Spearman’s r correlation coefficient, r = 0.285; p = 0.035), but not associated with other clinical parameters (Table 3). Overall, the PedsQL parent report scores were higher than the self-report scores.

### Cognitive ability evaluation

The scores of C-WISC were within the normal range. Children were divided into 2 groups: group A including pediatric uveitis with visual acuity equal to or better than 0.3 in their best eye (124 patients, 83.78%); group B with a vision less than 0.3 in their best eye (24 patients, 16.22%). Full-scale IQ and performance IQ were significantly lower in the group B as compared with the group A (p = 0.018; p = 0.005, Table 4). Intriguingly, verbal IQ of male children in the group B was significantly lower than that of male children in the group A (p = 0.016, Table 4). Spearman rank correlation test showed that performance IQ was significantly associated with the age at study participation (Spearman’s r correlation coefficient, r=-0.333; p = 0.017) and the age at uveitis diagnosis (Spearman’s r correlation coefficient, r=-0.284; p = 0.044) (Table 3).

**Table 4:** Scores for cognitive strengths and weaknesses using Chinese Wechsler Intelligence Scale for Children (C-WSIC) reported by male and female children

	N = 124	N = 24	*P* value
**Group**	A (BCVA ≥ 0.3)	B (BCVA < 0.3)	
**Score of Verbal IQ**			
Mean (SD)	112.18 (13.13)	106.92 (13.18)	0.178
§**Score of Performance IQ**			
Mean (SD)	88.08 (15.73)	70.17 (18.97)	0.005
§**Score of Full scale IQ**			
Mean (SD)	100.64 (13.66)	90.33 (9.04)	0.018
**Male children**			
	N = 63	N = 12	
**Group**	A (BCVA ≥ 0.3)	B (BCVA < 0.3)	
§**Score of Verbal IQ**			
Mean (SD)	113.71 (7.55)	103.43 (11.31)	0.016
**Score of Performance IQ**			
Mean (SD)	82.12 (14.98)	74.43 (7.57)	0.214
§**Score of Full scale IQ**			
Mean (SD)	98.94 (9.72)	89.00 (10.03)	0.034
**Female children**			
	N = 61	N = 12	
**Group**	A (BCVA ≥ 0.3)	B (BCVA < 0.3)	
**Score of Verbal IQ**			
Mean (SD)	111.00 (16.28)	111.8 (15.32)	0.921
§**Score of Performance IQ**			
Mean (SD)	92.68 (15.03)	64.20 (28.76)	0.028
**Score of Full scale IQ**			
Mean (SD)	101.95 (16.17)	92.20 (8.14)	0.206

Unpaired T test, P < 0.05

BCDVA = binocular corrected distant visual acuity; IQ = intelligence quotient; SD = standard deviation; §Statistically significant difference

### Anxiety and depression evaluation

As regard the HAD scores, the percentage of pediatric uveitis scored equal to or higher than 8 was 18.6% in depression scale and 21.8% in anxiety scale. There was no difference concerning the HAD scores for anxiety, depression and CES-DC scores between the group A and group B (p > 0.05, Table 5). Furthermore, Spearman rank correlation test showed that the CES-DC scores were positively associated with daily dose of cyclosporine used currently (Spearman’s r correlation coefficient, r = 0.309; p = 0.034). The HAD scores for anxiety and depression were not associated with any clinical parameters, such as BCVA, undergoing of surgery, medication used, ocular complications, or systemic signs (Table 3).

**Table 5:** Comparison of anxiety and depression in children with uveitis at different vision levels

	N = 131	N = 25	*P* value
**Group**	A (BCVA ≥ 0.3)	B (BCVA < 0.3)	
**Score of HAD for anxiety**			
Mean (SD)	4.42 (2.90)	4.36 (2.80)	0.958
**Score of HAD for depression**			
Mean (SD)	5.56 (3.28)	5.00 (2.90)	0.617
**Score of CES-DC**			
Mean (SD)	38.28 (7.26)	37.55 (4.59)	0.755

Mann-Whitney U test, P < 0.05

BCVA = binocular corrected visual acuity; HAD = Hospital Anxiety and Depression Scale; CES-DC = Center for Epidemiological Studies of Depression scale for Children; SD = standard deviation; §Statistically significant difference

### Parental education evaluation

The parental education information of children is listed in Table 6. On average, the parents of children with impaired vision in their best eye generally had lower levels of education than those of children with vision acuity equal to or better than 0.3 (p = 0.037).

**Table 6:** Parental education information of children with uveitis

	N = 131		N = 25	
**Group**	A (BCVA ≥ 0.3)		B (BCVA < 0.3)	
**Parental education**	n	%	n	%
Undergraduate degree	24	18.3	1	4.0
College degree	7	5.3	1	4.0
Polytechnic school	12	9.1	2	8.0
Senior high school	34	26.0	1	4.0
Junior high school	39	29.8	10	40.0
Primary school	15	11.5	10	40.0
*P* value	0.037			

## Discussion

In this study, we performed a comprehensive evaluation of functional visual ability, VR-QoL, HR-QOL, cognitive ability and depression and anxiety in children with uveitis. We found that pediatric uveitis had a significant impact on functional visual ability, VR-QoL, and HR-QoL. Furthermore, children with impaired vision had worse cognitive ability and the parents of children with impaired vision generally had lower levels of education.

Diseases occurring in children during their growth development period may have an impact on the formation of their emotional cognition and social function [1]. Children with uveitis have been shown to be associated with many ocular complications, such as synechia, cataract, band keratopathy, macular edema, etc. [4]. These complications as well as decreased vision may potentially affect visual-related, health-related quality of life and cognitive ability either due to disease itself or complications. A relatively large sample with wide range of patient sources in our uveitis center enable us to accurately evaluate the influences of pediatric uveitis on the quality of life, emotional state and cognitive ability.

The vision related functional ability and quality of life in children with uveitis have been assessed using the CVAQC and VR-QoL. Previous studies used both CVAQC and VR-QoL to assess the effects of ocular diseases on the functional visual ability and quality of life in children and adolescents [13, 14, 24]. The results showed that certain chronic ocular diseases had significant long-term effects on the quality of life, such as reading speed and education, due to impaired vision [25–27]. A recent clinical study showed that increased reading speed could improve QoL of patients with retinitis pigmentosa, and reading speed was the only modifiable factor affecting VR-QoL [28]. Another study indicated that patients with retinitis pigmentosa who had higher education levels showed better reading ability, and reduced visual acuity was a strong negative predictor of reading performance [29]. In this study, we used the above two scales to determine the vision-related quality of life in pediatric uveitis patients. The results showed that pediatric uveitis patients with higher scores of CVAQC and lower level of VR-QoL had more severe degree of visual impairment and poorer quality of life. This finding is consistent with previous studies [30].

Statement of HR-QoL has been evaluated by PedsQL self-report and PedsQL parental report. The scales are highly reliable and valid in children with various acute or chronic diseases including uveitis and glaucoma [31–33] [24, 34]. We used the HR-QoL to assess the health-related quality of life in children and their parents. The results showed that PedsQL self-reported scores were associated with systemic signs in children with uveitis, suggesting that concomitant systemic symptoms may severely affect children’s daily life. We also found that parents reported a greater impact of uveitis on HR-QoL than children themselves. In lines with our study, similar findings have been reported in children with cataract, glaucoma or juvenile idiopathic arthritis-associated uveitis [33, 35, 36] [24, 25]. This may be explained by that parents are more sensitive or nervous about their children’s ocular disorders than children themselves. It is interesting to note that the parents of children with impaired vision generally had lower educational attainment than those of children with better vision. This may be partially explained by early detection and timely intervention of children’s vision problems in a good educational family.

Previous studies reported that systemic diseases may affect the cognitive development of patients [37, 38]. However, there is little published data concerning whether visual impairment in pediatric uveitis could affect the cognitive development. In this study, we showed that performance IQ and full-scale IQ were profoundly affected by impaired vision in pediatric uveitis patients. Verbal IQ was significantly lower in male children with impaired vision, suggesting that the verbal related cognitive development is readily affected by decreased vision arising from pediatric uveitis. This result is, by and large, consistent with that reported previously [39, 40].

We also investigated anxiety and depression in pediatric uveitis using HAD scales. Although there was no significant difference in the HAD scores for anxiety and depression between children with impaired vision and those with a relatively better vision, the HAD scores for anxiety and depression were higher in pediatric uveitis as compared with those age-matched normal population reported previously [41, 42] [26, 27]. These results are generally consistent with those reported in adults with uveitis [11, 12]. Our findings suggest that uveitis children are also likely to have depressive or anxious emotional disorders, which may lead to thinking retardation, speech action reduction and activity decrease, and delayed development of children’s cognitive ability. In this study, we also showed that CES-DC scores were associated with daily dose of cyclosporine used concurrently. This result could be partially explained by central nervous side effects of this drug reported previously [43–45] [28–30]. Therefore, cyclosporine should be used carefully and minimally with particular attention paid to emotional monitoring during the treatment and management of pediatric uveitis.

This study has some limitations. First, subgroup analysis of pediatric uveitis according to uveitis entities or different visual impairment levels was not extensively performed in this study. Second, this study was performed only in children with uveitis, but not in age- and sex-matched healthy controls. The exact comparison concerning uveitis and normal children is expected to be carried out in future study. Third, specific uveitis PROMs for pediatric populations are needed in subsequent research. In addition, the impact of drug side effects on the cognitive ability and Qol of uveitis children would also be evaluated.

## Conclusion

In summary, this study evaluated FVA, VR-QoL, HR-QoL, cognitive ability as well as depression and anxiety in children with uveitis. Impaired vision had a significant impact on children’s FVA and QoL. In addition, uveitis children with poor vision are associated with impaired cognitive development. Prevention of visual impairment through early diagnosis, timely and properly treatment of pediatric uveitis may benefit the patients from improving functional vision quality of life and cognitive ability.

## Data Availability

The datasets used and/or analyzed during the current study available from the corresponding author on reasonable request.

## Abbreviations

BCVA: best-corrected visual acuity
logMAR: logarithm of the minimum angle of resolution
FVA: functional visual ability
CVAQC: Cardiff Visual Ability Questionnaire for Children
VR-QoL: vision-related quality of life
IVI-C: Impact of Vision Impairment for Children
HR-QoL: health-related quality of life
PedsQL: Pediatric Quality of Life Inventory
C-WISC: Chinese Wechsler Intelligence Scale for Children
HAD: Hospital Anxiety and Depression Scale

## References

[1] Shome A, Mugisho OO, Niederer RL, Rupenthal ID. Blocking the inflammasome: a novel approach to treat uveitis. Drug Discov Today 2021. 10.1016/j.drudis.2021.06.017 34229084

[2] Maleki A, Anesi SD, Look-Why S, Manhapra A, Foster CS. Pediatric uveitis: a comprehensive review. Surv Ophthalmol 2021. 10.1016/j.survophthal.2021.06.006 34181974

[3] Paivonsalo-Hietanen T, Tuominen J, Saari KM (2000). Uveitis in children: population-based study in Finland. Acta Ophthalmol Scand.

[4] Rosenberg KD, Feuer WJ, Davis JL (2004). Ocular complications of pediatric uveitis. Ophthalmology.

[5] Majumder PD, Biswas J (2013). Pediatric uveitis: an update. Oman J Ophthalmol.

[6] Chan NS, Choi J, Cheung CMG (2018). Pediatric Uveitis. Asia Pac J Ophthalmol (Phila).

[7] Angeles-Han ST (2015). Quality-of-life metrics in pediatric uveitis. Int Ophthalmol Clin.

[8] Taha R, Papadopoulou M, Zetterberg M, Oskarsdottir S, Gronlund MA (2019). Visual function and quality of Life in a cohort of Swedish Children with Juvenile Idiopathic Arthritis. Clin Ophthalmol.

[9] Ezzahri M, Amine B, Rostom S, Rifay Y, Badri D, Mawani N, Gueddari S, Shyen S, Wabi M, Moussa F (2013). The uveitis and its relationship with disease activity and quality of life in moroccan children with juvenile idiopathic arthritis. Clin Rheumatol.

[10] McDonald J, Cassedy A, Altaye M, Andringa J, Cooper AM, Drews-Botsch C, Engelhard G Jr, Hennard T, Holland GN, Jenkins K et al. Comprehensive assessment of quality of life, functioning and mental health in children with juvenile idiopathic arthritis and non-infectious uveitis. Arthritis Care Res (Hoboken) 2021. 10.1002/acr.24551 PMC8267048 33421338

[11] Silva LMP, Arantes TE, Casaroli-Marano R, Vaz T, Belfort R, Muccioli C (2019). Quality of life and psychological aspects in patients with visual impairment secondary to Uveitis: a clinical study in a Tertiary Care Hospital in Brazil. Ocul Immunol Inflamm.

[12] Onal S, Oray M, Yasa C, Akman M, Uludag G, Koc Akbay A, Tugal-Tutkun I (2018). Screening for depression and anxiety in patients with active Uveitis. Ocul Immunol Inflamm.

[13] Daiber HF, Gnugnoli DM. Visual acuity. StatPearls. edn. Treasure Island (FL); 2022.

[14] Khadka J, Ryan B, Margrain TH, Court H, Woodhouse JM (2010). Development of the 25-item Cardiff Visual ability questionnaire for children (CVAQC). Br J Ophthalmol.

[15] Huang J, Khadka J, Gao R, Zhang S, Dong W, Bao F, Chen H, Wang Q, Chen H, Pesudovs K (2017). Validation of an instrument to assess visual ability in children with visual impairment in China. Br J Ophthalmol.

[16] Cochrane GM, Marella M, Keeffe JE, Lamoureux EL (2011). The impact of Vision Impairment for Children (IVI_C): validation of a vision-specific pediatric quality-of-life questionnaire using rasch analysis. Invest Ophthalmol Vis Sci.

[17] Varni JW, Seid M, Knight TS, Uzark K, Szer IS (2002). The PedsQL 4.0 generic core scales: sensitivity, responsiveness, and impact on clinical decision-making. J Behav Med.

[18] Varni JW, Seid M, Kurtin PS (2001). PedsQL 4.0: reliability and validity of the Pediatric Quality of Life Inventory version 4.0 generic core scales in healthy and patient populations. Med Care.

[19] Lichtenberger EO (2005). General measures of cognition for the preschool child. Ment Retard Dev Disabil Res Rev.

[20] Snaith RP (2003). The hospital anxiety and Depression Scale. Health Qual Life Outcomes.

[21] Cochrane G, Lamoureux E, Keeffe J (2008). Defining the content for a new quality of life questionnaire for students with low vision (the impact of Vision Impairment on Children: IVI_C). Ophthalmic Epidemiol.

[22] Varni JW, Burwinkle TM, Seid M (2006). The PedsQL 4.0 as a school population health measure: feasibility, reliability, and validity. Qual Life Res.

[23] Varni JW, Burwinkle TM (2006). The PedsQL as a patient-reported outcome in children and adolescents with Attention-Deficit/Hyperactivity disorder: a population-based study. Health Qual Life Outcomes.

[24] Morthen MK, Magno MS, Utheim TP, Snieder H, Jansonius N, Hammond CJ, Vehof J (2022). The vision-related burden of dry eye. Ocul Surf.

[25] Vashist P, Gupta, Noopur T, Radhika G, Dwivedi SK (2016). Sadanand, Mani, Kalaivani: Population-based assessment of vision-related quality of life in corneal disease: results from the CORE study. Br J Ophthalmol.

[26] Zhang Y, Lin, Tong, Jiang A, Zhao, Naiqing, Gong L (2016). Vision-related quality of life and psychological status in chinese women with Sjogren’s syndrome dry eye: a case-control study. BMC Womens Health.

[27] Tailor VK, Abou-Rayyah Y, Brookes J, Khaw PT, Papadopoulos M, Adams GGW, Bunce C, Dahlmann-Noor A (2017). Quality of life and functional vision in children treated for cataract-a cross-sectional study. Eye (Lond).

[28] Altinbay D, Taskin I (2021). Evaluation of vision-related quality of life in retinitis pigmentosa patients with low vision. Jpn J Ophthalmol.

[29] Virgili G (2004). Reading performance in patients with retinitis pigmentosa: a study using the MNREAD charts. Invest Ophthalmol Vis Sci.

[30] AlDarrab A, Qurashi A, Thiabi MA (2019). Saad, Khandekar, Rajiv, Edward, Deepak P: functional visual ability and quality of life in children with Glaucoma. Am J Ophthalmol.

[31] Angeles-Han ST, Yeh S, McCracken C, Jenkins K, Stryker D, Myoung E, Vogler LB, Rouster-Stevens K, Lambert SR, Harrison MJ (2015). Using the Effects of Youngsters’ eyesight on Quality of Life Questionnaire to measure visual outcomes in children with Uveitis. Arthritis Care Res (Hoboken).

[32] Senthil MP, Simon S, Constable PA (2023). A review of patient-reported outcome measures used in uveitis. Surv Ophthalmol.

[33] Dahlmann-Noor A, Tailor V, Bunce C, Abou-Rayyah Y, Adams G, Brookes J, Khaw PT, Papadopoulos M (2017). Quality of life and functional vision in children with Glaucoma. Ophthalmology.

[34] Stadler C, Bolten M, Schmeck K (2011). Pharmacotherapeutic intervention in impulsive preschool children: the need for a comprehensive therapeutic approach. Child Adolesc Psychiatry Ment Health.

[35] Sestan M (2020). Quality of life in children suffering from juvenile idiopathic arthritis-associated uveitis. Rheumatol Int.

[36] Chak M, Rahi JS, British Congenital Cataract Interest G (2007). The health-related quality of life of children with congenital cataract: findings of the british congenital cataract study. Br J Ophthalmol.

[37] Ma Y, Chen G, Wang Y, Xu K (2015). Language dysfunction is associated with age of onset of benign epilepsy with centrotemporal spikes in children. Eur Neurol.

[38] Bai Y, Gao MY. Effect of crawling training on the cognitive function of children with cerebral palsy. Int J Rehabil Res 2022. 10.1097/MRR.0000000000000526 35347101

[39] Schumann CM, Julia H, Goodlin-Jones BL, Kwon, Hower R, Allan L (2007). Amaral, David G: hippocampal size positively correlates with verbal IQ in male children. Hippocampus.

[40] Tallal P (1991). Hormonal influences in developmental learning disabilities. Psychoneuroendocrinology.

[41] Barker MM, Beresford B, Bland M, Fraser LK (2019). Prevalence and incidence of anxiety and depression among children, adolescents, and young adults with life-limiting conditions: a systematic review and Meta-analysis. JAMA Pediatr.

[42] Ling Y, Liu C, Scott Huebner E, Zeng Y, Zhao N, Li Z (2021). A study on classification features of depressive symptoms in adolescents. J Ment Health.

[43] Mesripour A, Golbidi M, Hajhashemi V (2020). Dextromethorphan improved cyclosporine-induced depression in mice model of despair. Res Pharm Sci.

[44] Trzepacz PT, Levenson JL, Tringali RA (1991). Psychopharmacology and neuropsychiatric syndromes in organ transplantation. Gen Hosp Psychiatry.

[45] Thompson CB, June CH, Sullivan KM, Thomas ED (1984). Association between cyclosporin neurotoxicity and hypomagnesaemia. Lancet.

